# Three-dimensional models: from cell culture to Patient-Derived Organoid and its application to future liposarcoma research

**DOI:** 10.32604/or.2024.053635

**Published:** 2024-12-20

**Authors:** SAYUMI TAHARA, SYDNEY RENTSCH, FERNANDA COSTAS CASAL DE FARIA, PATRICIA SARCHET, ROMA KARNA, FEDERICA CALORE, RAPHAEL E. POLLOCK

**Affiliations:** 1 Department of Surgery, Division of Surgical Oncology, The James Comprehensive Cancer Center, The Ohio State University Wexner Medical Center, Columbus, OH 43210, USA; 2 Department of Cancer Biology and Genetics, The James Comprehensive Cancer Center, The Ohio State University Wexner Medical Center, Columbus, OH 43210, USA

**Keywords:** Cell culture, Liposarcoma, Patient-Derived Organoid (PDO), Spheroid, Three-dimensional (3D) cell culture

## Abstract

Liposarcoma is one of the most common soft tissue sarcomas, however, its occurrence rate is still rare compared to other cancers. Due to its rarity, *in vitro* experiments are an essential approach to elucidate liposarcoma pathobiology. Conventional cell culture-based research (2D cell culture) is still playing a pivotal role, while several shortcomings have been recently under discussion. *In vivo*, mouse models are usually adopted for pre-clinical analyses with expectations to overcome the issues of 2D cell culture. However, they do not fully recapitulate human dedifferentiated liposarcoma (DDLPS) characteristics. Therefore, three-dimensional (3D) culture systems have been the recent research focus in the cell biology field with the expectation to overcome at the same time the disadvantages of 2D cell culture and *in vivo* animal models and fill in the gap between them. Given the liposarcoma rarity, we believe that 3D cell culture techniques, including 3D cell cultures/co-cultures, and Patient-Derived tumor Organoids (PDOs), represent a promising approach to facilitate liposarcoma investigation and elucidate its molecular mechanisms and effective therapy development. In this review, we first provide a general overview of 3D cell cultures compared to 2D cell cultures. We then focus on one of the recent 3D cell culture applications, Patient-Derived Organoids (PDOs), summarizing and discussing several PDO methodologies. Finally, we discuss the current and future applications of PDOs to sarcoma, particularly in the field of liposarcoma.

## Introduction

Liposarcoma (LPS) is the most common histological type of soft tissue sarcoma, encompassing five subtypes based on WHO classification of tumors, 5th edition: atypical lipomatous tumor/well-differentiated liposarcoma (ALT/WDLPS), dedifferentiated liposarcoma (DDLPS), myxoid liposarcoma (MLPS), pleomorphic liposarcoma and myxoid pleomorphic liposarcoma [1] (Table 1).

**Table 1:** Liposarcoma classification

	Localization	Epidemiology	Pathogenesis	Histopathology	Diagnostic molecular pathology	Prognosis
ALT/WDLPS	Deep soft tissue of proximal extremities and trunk. The retroperitoneum and the paratesticular area are also involved.	The largest subgroup of adipocytic malignancies accounts for approximately 40–45% of all liposarcomas.	Supernumerary rings and giant markers contain amplified sequences originating from the 12q14-q15 region. MDM2 is the main driver gene. Several other genes in the 12q14-q15 region, including TSPAN31, CDK4, HMGA2, YEATS4, CPM, and FRS2, are frequently coamplified with MDM2.	Can be subdivided into three main subtypes: adipocytic (1), sclerosing (2), and inflammatory (3).	Detection of MDM2 and/or CDK4 amplification.	No potential for metastasis unless it undergoes dedifferentiation.
				Mature adipocytes in which substantial variation in cell size and nuclear atypia (1), with a background of an extensive fibrillary collagenous stroma (2) or a chronic inflammatory infiltrate (3).		
DDLPS	Retroperitoneum is the most common. Other locations include the spermatic cord, mediastinum, head and neck, and trunk.	Dedifferentiation occurs in as many as 10% of WDLPSs, but the risk can be higher for deep-seated lesions.	Overlaps with ALT/WDLPS. In addition, amplification of JUN, TERT, CPM, MAP3K5, and other genes from the 6q12-q24 region can be observed.	A transition from ALT/WDLPS to non-lipogenic sarcoma. Dedifferentiated areas most frequently resemble undifferentiated pleomorphic sarcoma or myxofibrosarcoma.	Detection of MDM2 and/or CDK4 amplification.	Local recurrence rate is at least 40%, distant metastases are observed in 15–20% of cases, with an overall mortality rate of 28–30% at 5-year follow-up.
MLPS	Deep extremities, most often the thigh.	Approximately 20–30% of liposarcomas and 5% of adult soft tissue sarcomas.	*t* (12;16) (q13; p11) translocation generating FUS-DDIT3 fusion transcrips.	MLPS typically lacks atypia, substantial mitotic activity, or spindling. The tumors contain abundant, lightly basophilic, myxoid stroma, but it is diminished in high-grade MLPS. Immunohistochemistry plays little role in the diagnosis of MLPS.	Demonstration of the translocation/fusion transcript may help distinguish MLPS from other myxoid sarcomas. FUS and EWSR1 can substitute for each other and occur in other sarcomas, whereas DDIT3 is unique to MLPS.	The local recurrence rate is in approximately 12–25% of cases. MLPS often metastasize to other soft tissue sites and bone, distant metastases are observed in 30–60% of cases.
Pleomorphic liposarcoma	The extremities in two-thirds of cases, following the trunk wall, retroperitoneum, and spermatic cord.	A rare subtype, <5% of all liposarcoma.	More closely resembles other pleomorphic sarcomas than ALT/WDLPS, DDLPS, or MLPS. No pathognomonic structural rearrangement, such as recurrent translocation or consistent presence of supernumerary ring chromosomes, has not been identified.	All tumors contain a varying proportion of pleomorphic lipoblasts, with infiltrative margins. Epithelioid morphology is seen in about one-quarter of cases.	The absence of amplification of MDM2 can help distinguish pleomorphic liposarcoma from DDLPS.	An aggressive tumor exhibiting local recurrence and metastatic rates of 30–50%. Overall 5-year survival rate of about 60%. Metastases occur mostly in the lungs and pleura.
				MDM2 and/or CDK4 staining typically shows negative. The epithelioid subtype may be positive for keratins and melan-A.		
Myxoid pleomorphic liposarcoma	A predilection for the mediastinum, but thigh, head and neck, perineum, abdomen, and back were also reported.	Predominantly in children and young adults.	Associated with Li-Fraumeni syndrome, numerical chromosomal aberration, and inactivation of the RB1 tumor suppressor gene.	The tumor shows variable proportions of myxoid liposarcoma-like areas with the background of lymphangioma-like myxoid pools. A progressive transition into high-grade pleomorphic liposarcoma-like areas may be also observed. No specific immunophenotype is useful for immunohistochemistry.	Lacks of FUS/EWSR1-DDIT3 gene fusions (seen in MLPS) or MDM2 amplification (seen in WDLPS/DDLPS).	An extremely aggressive tumor with a high recurrence rate and poor overall survival, metastasis to lung, bone, and soft tissue.

Note: Summary of localization, epidemiology, pathogenesis, histopathology, diagnostic molecular pathology, and prognosis for each group.

ALT/WDLPS represents the largest subgroup of adipocytic malignancies, accounting for approximately 40%–50% of all liposarcomas. DDLPS is another common form, occurring in up to 10% of WDLPS cases. DDLPS shares a similar patient population with ALT/WDLPS, including peak incidence in middle-aged adults, equal occurrence rates between males and females, a deep-seated location particularly in the retroperitoneum, and a painless mass. The pathogenesis of DDLPS overlaps with that of ALT/WDLPS, with consistent amplification of *MDM2* and/or *CDK4* (12q13-q15) [2,3].

In contrast, MLPS and its hypercellular subtype, formerly known as round cell liposarcoma, are typically present within deep soft tissues of the extremities, most often the thigh, and are characterized by the *t* (12;16) (q13; p11) translocation, generating FUS-DDIT3 fusion transcripts. Pleomorphic liposarcoma and myxoid pleomorphic liposarcoma are rare subtypes with aggressive behavior, lacking the specific gene fusions and amplifications observed in ATL/WDLPS, DDLPS, and MLPS.

While surgical-wide excision remains the mainstay treatment for liposarcoma, complete resection is the only approach for a radical cure. Several treatments have been attempted in clinical settings, including cytotoxic chemotherapy agents such as doxorubicin, ifosfamide, gemcitabine, and docetaxel, which have shown some efficacy in unselected patient populations [4]. Recently, several targeted therapy drugs have been proposed as potentially effective treatments for LPS. Given that some LPS subtypes display specific aberrations, such as the 12q13-15 amplicons leading to the amplification of MDM2 and CDK4 in WDLPS/DDLPS and FUS-DDIT3/EWSR1-DDIT3 fusion in MLPS, these genomic alterations could represent potential therapeutic targets. MDM2 antagonists, such as RG7388 and Nutlin 3A, and CDK4/6 inhibitors (palbociclib, ribociclib, abemaciclib, and TQB3616), are either clinically-used drugs or currently under clinical trials [5]. These treatments hold the potential for effective therapy for LPS, both as single treatments and in combination [6,7].

Immune-checkpoint therapy is also being explored. Roland et al. reported a phase-2 study of neoadjuvant immune-checkpoint blockade (ICB) in dedifferentiated liposarcoma [8]. This study observed an increase in B cell infiltration into the tumor following neoadjuvant treatment, which was associated with overall survival. They concluded that neoadjuvant ICB is associated with complex immune changes within the tumor microenvironment in DDLPS. Given the limited studies on immune checkpoint therapy, the work of Roland and colleagues provides valuable insights for developing successful liposarcoma therapies.

**Figure 1:**
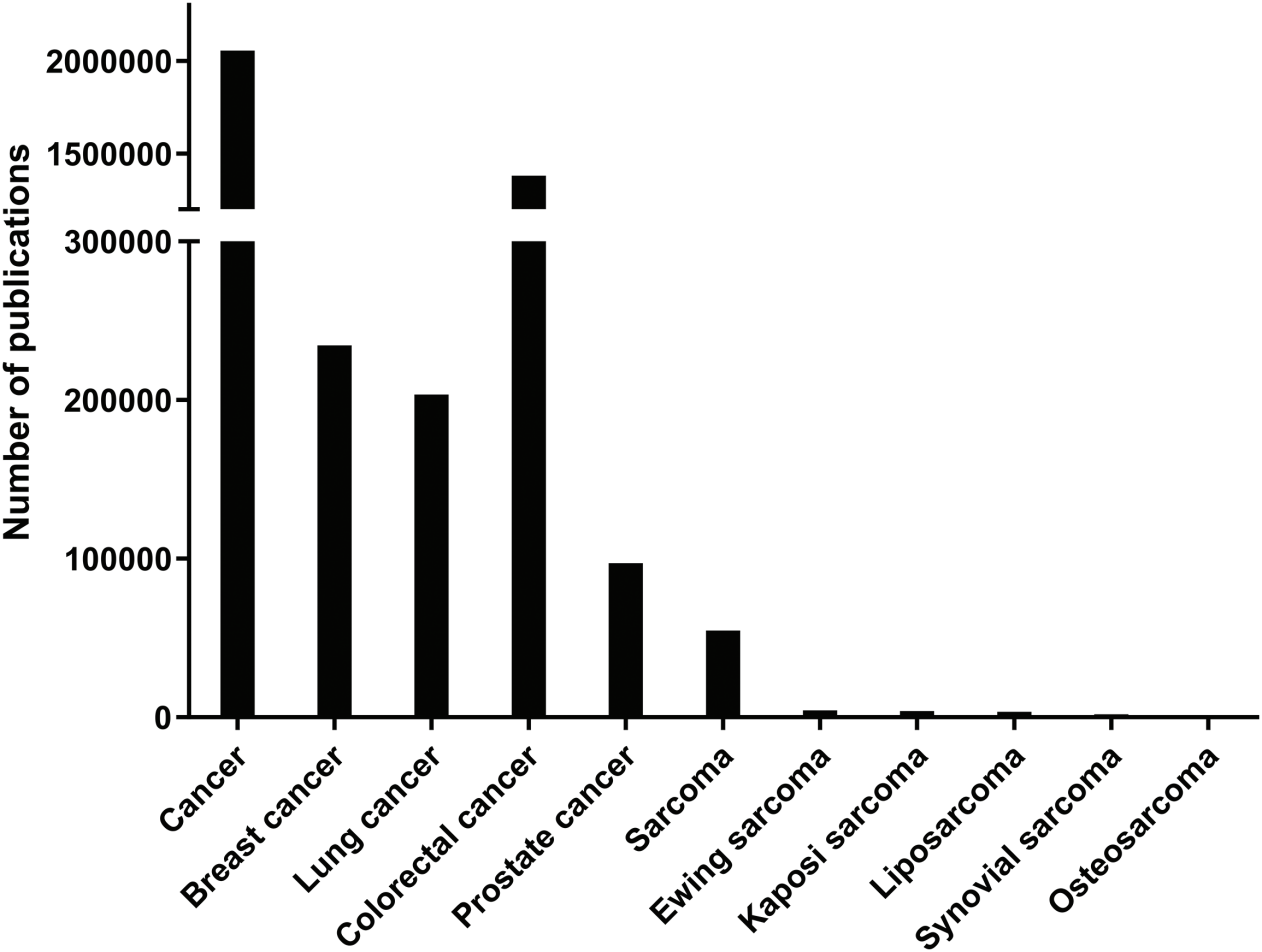
Number of publications for each category in PubMed from 2014 to 2024. The number of indexed PubMed publications containing each category which is indicated in *X*-axis. There are significantly fewer publications related to sarcomas compared to epithelial cancers.

Although liposarcoma is one of the most common soft tissue sarcomas, its occurrence rate is still rare compared to other cancers; indeed, soft tissue sarcomas account for less than 1% of tumors in adults [9]. According to the most searched studies on PubMed (https://pubmed.ncbi.nlm.nih.gov, accessed on 1 May 2024) in the last 10 years, the number of accessible articles related to “cancer” was 2,040,914, “breast cancer” was 232,974, “lung cancer” was 201,791, and “colorectal cancer” was 138,842. In contrast, “sarcoma” related articles numbered just 54,628, and “liposarcoma” only 3470 (Fig. 1).

Due to its rarity, *in vitro* experiments are essential for elucidating liposarcoma pathobiology. Cell culture studies remain pivotal *in vitro* investigation as the most common for understanding sarcoma mechanisms. Conventional cell culture-based research (two-dimensional cell culture, 2D cell culture) offers advantages such as wide availability, ease of handling, high reproducibility, and low cost [10,11]. However, several shortcomings have been discussed recently: failure to mimic the heterogeneity of original tumors or *in vivo* microenvironment, and the possibility of substantial and unpredictable genetic changes after several passages [12,13].

*In vivo*, mouse models are usually adopted for pre-clinical analyses to overcome the issues of 2D cell culture, but the number of transgenic mouse models developed for liposarcoma is limited. There are two genetic models of DDLPS [14,15], however, they do not fully recapitulate the complexity, heterogeneity, and characteristics of human DDLPS. Patient-derived xenografts (PDXs) may undergo mouse-specific evolution [16]. Moreover, they are very resource- and time-consuming to develop, and their use is limited by feasibility and ethical issues [12]. Therefore, three-dimensional (3D) cell culture systems have been a recent research focus in cell biology, with the expectation of overcoming the disadvantages of 2D cell culture and *in vivo* animal models, bridging the gap between them. The ability to investigate cell-cell or cell-matrix interaction *in vitro* using patient-derived resources is a significant advantage. Given the rarity of LPS, we believe that 3D cell culture techniques, including 3D cell cultures/co-cultures, and Patient-Derived tumor Organoids (PDOs), represent a promising approach to facilitate liposarcoma investigation and elucidate its molecular mechanisms and effective therapy development. Furthermore, we believe that liposarcoma 3D model-based research should be encouraged, given the very few reports published so far. As mentioned above, different chemotherapies and radiotherapy are currently underway, but further improvements are still required.

In this review, we aim to introduce the fundamental knowledge about 3D cell cultures, including their advantages and disadvantages, and their classification. Then, we focus on one of the recent 3D cell culture applications, PDOs. Finally, we aim to discuss the application of PDOs to sarcoma, specifically liposarcoma, for future research.

## 2D *vs.* 3D Cell Culture

### Comparison between 2D and 3D cell culture

For more accurate and clinically applicable cancer research, tumor cells should ideally be grown in an environment that closely mimics their physiological conditions and structure. Conventional 2D cell culture has limitations in replicating physiological conditions, such as a lack of 3D structures, cell-cell or cell-matrix interactions, and heterogeneity. These shortcomings arise from the 2D environment, which does not effectively replicate tumor structure. However, these limitations can be overcome by using 3D cell cultures [17].

3D cell cultures better mimic natural tissue or organ structures and preserve their morphology, hence allowing the study of molecular mechanisms regulating tumor growth [18–21], *in vivo* like cell-cell and cell-ECM interactions [22], and can preserve tumor heterogeneity [23]. This leads to good reproducibility of physiological conditions. However, long-time cultivation, higher costs [10,24], and more elaborated protocols [25] could represent a disadvantage for 3D-based studies.

### 3D cell culture methods: scaffold-based and scaffold-free

Various 3D cell culture techniques have been developed to better recapitulate the *in vivo* cancer condition. 3D culture systems can be broadly divided into two categories: scaffold-based techniques and scaffold-free techniques. Hydrogels, used as scaffolds, can be further classified into several groups based on their sources, compositions, crosslinking, configuration, ionic charge, properties, response, and more [26]. Choosing a specific scaffold from over 100 types of natural or synthetic ones to elicit a particular type of morphological and physiological behavior in cultured cells. Therefore, a better understanding of scaffold characteristics is needed for an ideal 3D cell culture design for better investigations. In this section, we will discuss natural scaffold-based and scaffold-free 3D cell cultures.

## Natural Scaffold-Based 3D Cell Culture

Collagen [27,28], fibrin, and hyaluronic acid [27] are some well-used natural scaffold biomaterials, but other naturally-derived materials such as silk [29], gelatin [30], and alginate [31] can be also included in this category.

Collagen is widely utilized in 3D cell culture due to its unique characteristics, which provide a suitable microenvironment for the specific functions and properties of tissues [32]. For example, the architecture of collagen hydrogels can be controlled by manipulating ionic force, pH, and temperature, affecting the scaffold’s mechanical properties, architecture, and biodegradability, which in turn influences fiber thickness and pore size of the gels [33]. Silk could be integrated with collagen when stiffer hydrogels are required, especially in tissue engineering [34–36]: Sanz-Fraile et al. optimized this integration to obtain a useful bioink for 3D bioprinting hydrogel scaffolds [29]. Hydrogels can have different properties in terms of stiffness, density, composition, permeability, and degradability, all of which may profoundly affect cell activity, migration, adhesion, proliferation, and differentiation.

Matrigel, a complex mixture of multiple proteins and associated molecules, is an alternative to simplified single components of the extracellular matrix (ECM). Matrigel is a reconstituted basement membrane derived from extracts of Engelbreth-Holm-Swarm mouse tumors, containing abundant ECM components such as collagen type IV, entactin, perlecan, laminin, and several growth factors or metalloproteinases [37–39]. Matrigel has been reported to promote the differentiation and outgrowth of differentiated cells from tissue explants, using many different cell types such as hepatocytes [40,41], breast cancer cells [42], and Sertoli cells [43].

Although both natural and synthetic polymer-based scaffolds have various advantages such as high mechanical properties, processibility, and stability, natural polymer-based hydrogels are regarded as superior to synthetic ones in terms of bioactive properties such as versatility, biocompatibility and degradability *in vivo* [44]. Natural polymers perform a diverse set of functions, such as promoting cell-cell interactions and thus enhancing tissue performance [45]. Moreover, natural polymers can more easily incorporate cell membrane receptors and peptide ligands, facilitating the adhesion, spreading, and growth of cells within the hydrogel matrix. Another characteristic of natural polymeric hydrogels is their ability to hold cells and drugs in their structure and deliver them to specific sites in a controlled manner, promising broad applications in biomedical fields [26].

Methodologically, cells are seeded on top of gelled material [46,47], or mixed with the matrix before it solidifies [28,48–50] in general. Once the gels are solidified, culture media is added. Media is typically changed every 2 or 3 days and cells are cultivated in this manner until the experiment is terminated. Depending on the experimental endpoints and goals, minor adjustments to the protocol may be necessary.

## Scaffold-Free 3D Cell Culture

In scaffold-free systems, cells are encouraged to aggregate in their culture media following gravity. The constructs are generally called spheroids due to their spherical shape. Spheroids are produced in multiple ways, using alternative materials [51]. For example, the hanging drop technique is one of the simplest and most utilized methods: after the cell pellet is resuspended in culture media, small droplets containing single cells are generated on a plate. The plate is then immediately flipped over, preventing cells from attaching to the plastic bottom and keeping them suspended within the droplet. Gravity pulls cells down, facilitating cell aggregation within the droplets and leading to spheroid formation. Cost efficacy and multiple application options are the advantages of this method, while the need for an extra spheroid transfer step for further analysis and difficulty in changing media are some disadvantages [25].

However, some researchers have reported strategies to overcome these disadvantages. For example, Foty transferred sheet-like structures obtained 18 h after cell seeding to round-bottom glass shaker flasks containing a complete medium, then incubated the structures in a shaking water bath at 37°C [52]. This method made it possible to conduct drug treatment studies using hanging drop spheroids. Huang et al. invented a microfluidic-based hanging-drop culture system, which made a medium change for hanging drop spheroids accessible [53]. They developed two types of devices: a cylindrical tube microfluid chip and a taper-shaped microfluid chip, both superior to the conventional hanging drop method in terms of droplet stability, spheroid formation, and longer-term culture.

The spheroid formation is also achievable using low-adherence substrates, which minimize cell attachment to the culture plate surface, allowing cells to spontaneously aggregate at the bottom of wells. Using this system, media change or drug treatment can be simply performed in the same plate by aspirating or adding a culture medium, eliminating the need for an extra transfer process. Co-culture using ultralow attachment plates has already been reported: Jang et al. seeded two different cell lines, human dermal papilla (DP) and human outer root sheath (ORS) cells, for alopecia research, using ultralow attachment plates. Co-cultured cells, seeded at different time points, formed two-layered spheroids mimicking the hair follicle structure and displayed spherical shape elongation due to cell proliferation [54].

Thakur et al. generated spheroids from Wharton’s jelly mesenchymal stem or stromal cells (WJ-MSCs) using three different scaffold-free methods: 3D micro-well, hanging drop, and ultra-low attachment plate and compared them to conventional 2D cell culture models [55]. Interestingly, the mRNA expression of senescence-related markers such as p16, p21, and p53 was decreased in the 3D cultured cells compared to 2D cultured cells, while the expression of BCLX, an anti-apoptotic factor, was significantly increased in 3D-cultured cells. Moreover, mRNA expression of immunomodulatory/anti-inflammatory factors, such as Indoleamine 2,3-dioxygenase (IDO), Interleukin-10 (IL-10), Leukaemia inhibitory factor (LIF), Angiotensin1, and Vascular endothelial growth factor (VEGF), was increased in the 3D cultured WJ-MSCs compared to the conventional cell culture counterpart, suggesting that cells cultured in 3D would be more effective in reducing inflammatory responses or modulating immune responses than 2D- cultured cells [55].

The spinner culture technique is another scaffold-free 3D cell culture method introduced by Habanjar et al. [56]. In this technique, the cell suspension is continuously mixed in a specific spinner centrifugal flask bioreactor controlled by convective forces generated by an impeller or magnetic stir bar. This constant stirring promotes spheroid formation. One advantage is that fluid movement supports high throughput [57]. However, there are also some disadvantages: a cell type with lower cohesion might not be suitable, too high a stirring speed might cause spheroid disruption or cell damage, and it is hard to monitor spheroid formation.

The rotating wall vessel technique utilizes constant rotation to facilitate spheroid formation [58]. In this method, the culture dish is rotated, keeping cells continuously in a suspended state without the turbulence that could be generated by conventional stirred bioreactors [59]. The microgravity caused by this suspended condition affects gene expression of mesenchymal stem cells, reducing chondrogenic and osteogenic gene expression while elevating adipogenic gene expression [60].

Lastly, the application of microgravity to cell culture is an interesting topic. Microgravity during space flight leads to physiological and anatomical changes in an astronaut’s body [61], including bone atrophy [62], muscle atrophy [63], and cardiovascular deconditioning [64,65], as well as dynamic changes at the cellular and molecular levels [66]. A specific device is needed to create a microgravity condition, which could be a disadvantage. However, under-stimulated microgravity, the attenuation of cell differentiation was reported using rat myoblast cells [67] and rat bone marrow mesenchymal stem cells [68], and spheroid formation was also confirmed [69].

## Patient-Derived Organoids (PDOs) and Their Applications

**Figure 2:**
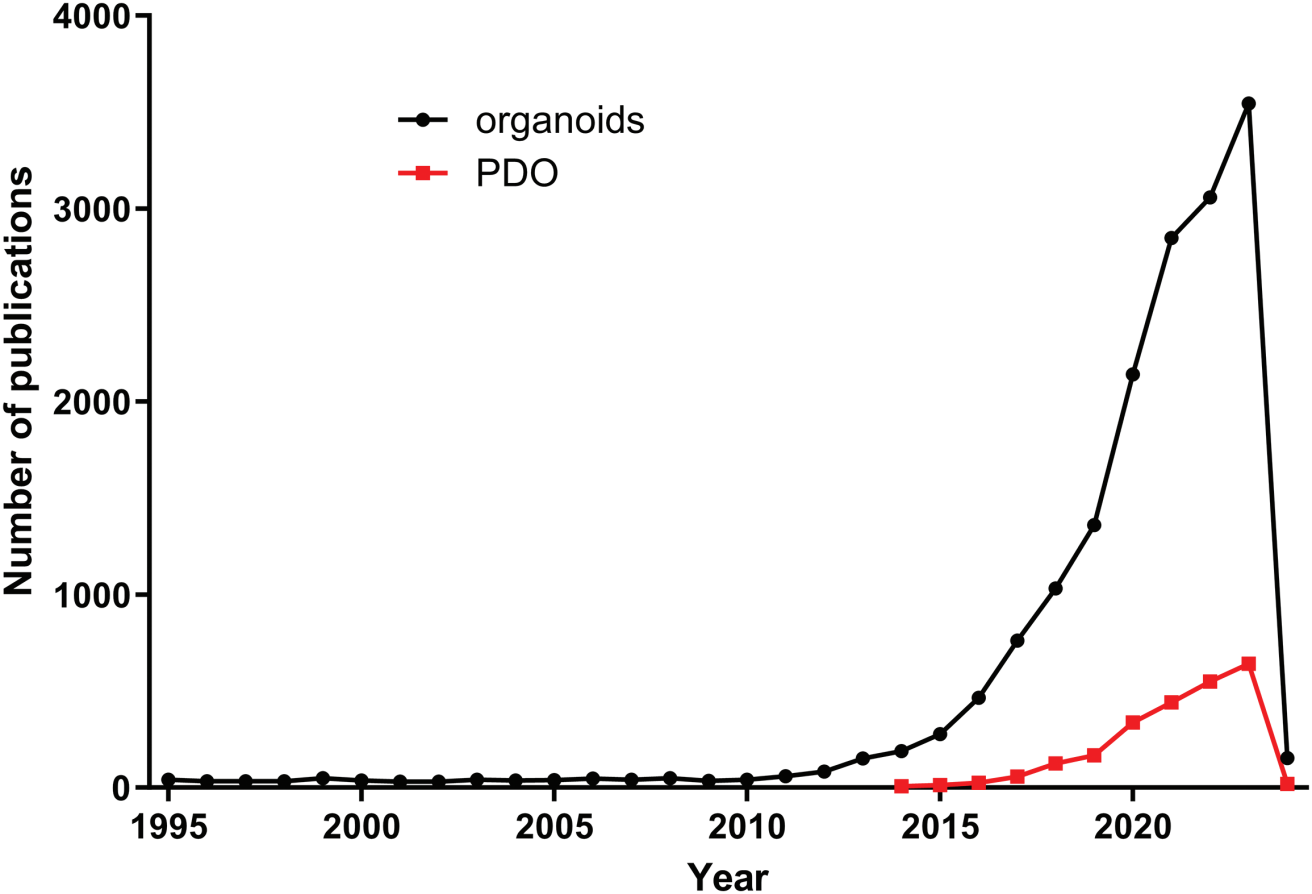
Comparison of number of publications on organoids *vs*. PDOs. Organoid-related publications have increased since 2010, while PDO-related publications stared to rise from 2014.

Organoids are *in vitro* models that mimic *in vivo* conditions, capable of forming 3D structures under certain ECM parameters. In an organoid model, cells can organize themselves to resemble the original organ or tumor in both structure and function. There are two types of organoids, classified by their origin: pluripotent stem cell-derived or adult stem cell-derived [70,71]. PDOs, discussed in this review, fall into the latter category. While research using organoids began around the year 2000 [72] and continues to show an increase in the number of publications, the number of PDO reports has been rising since 2014 based on PubMed searches (Fig. 2).

Most PDO reports so far have focused on epithelial cancer research, such as gastrointestinal cancer [73–75], cholangiocarcinoma [76], lung cancer [77,78], prostate cancer [79], endometrial cancer [80], ovarian cancer [81], and bladder cancer [82]. Additionally, some papers have investigated brain tumors [83], particularly glioblastoma [84]. Applications of PDOs are extensive, including establishment and characterization compared to the original specimen, histopathological analysis, next-generation sequencing, drug screening, and predicting resistance to neoadjuvant chemotherapy.

For example, a recent study by Thorel et al. performed multiple analyses on PDO models. The authors established seven different models from the same Ovarian Clear Cell Carcinoma (OCCC) patient tissue: four cell lines, two Patient-Derived Tumor Organoids (PDTOs), and one patient-derived xenograft (PDX). They conducted comprehensive characterizations based on morphological, histological, and transcriptomic analyses, as well as evaluations of responses to treatments administered to the patient [85]. The original tumor displayed large, poorly differentiated tumor cells with protrusion of hobnail cells, characteristic of OCCC. The PDX model closely resembled the patient’s original tumor, while the PDTO models showed a more differentiated architecture with hobnail cells and the formation of tubular patterns. Three out of four cell lines showed an epithelial phenotype, while the last one showed a mesenchymal-like phenotype. The authors treated all models with carboplatin, doxorubicin, and gemcitabine: PDX and PDTO models displayed resistance to the three treatments, similar to the patient’s response, while the four cell lines showed higher sensitivity to the drugs. These findings suggest that PDTOs are more similar to the PDX model in terms of *in vivo* condition reproducibility, making drug response results from PDO models potentially more reliable than those from conventionally cultured cell lines.

Another example of the reliability of PDO models was provided by Jiang et al., who established PDOs from bladder cancer and its metastatic ascites. The authors treated PDOs with cisplatin, carboplatin, oxaliplatin, and olaparib, and reported that PDOs showed different sensitivities to these drugs, indicating the potential of PDOs to facilitate the development of personalized medicine [82]. Moreover, Papaccio et al. used 29 PDO lines established from patient-derived colorectal cancer tissue to perform targeted next-generation sequencing (NGS), copy number variation analysis, RNA-sequencing analysis, and proteomic analysis, and treated them with both standard and non-standard agents [74]. PDOs successfully recapitulated not only the morphology and protein expression patterns of corresponding tissues but also the genomic and transcriptomic profiles of the original tissues. Moreover, the authors analyzed drug efficacy on PDOs compared to the response of matched patients by treating PDOs with the same drug administered to the matched patient clinically. Consequently, one PDO showed corresponding sensitivity to the patient, while another showed enhanced sensitivity compared to the patient’s reaction. Therefore, the authors could not conclusively prove that PDOs can accurately recapitulate patient responses to treatment in this study. However, they concluded that the organoid line derived from the primary tumor should be considered in the context of translational studies.

## PDO Establishment Methods

In this section, we will focus on PDO generation methods and their comparison. Although protocols may differ depending on the tissue origin or research focus, most PDO cultures can be conducted using the major technique of natural scaffold-based 3D cell culture methods. Additional minor modifications may be employed to optimize each protocol. Most protocols developed to generate PDOs from primary patient materials typically require tissue digestion before cell seeding, although some reports suggest tissue digestion is not always necessary. Collagenase is one of the most popular enzymes used for tissue digestion, but it may be replaced with different enzymes depending on the downstream research goals or the type of primary tissue. DNase, trypsin, hyaluronidase and ethylenediaminetetraacetate (EDTA) are candidate components that could be added to collagenase or used as alternatives [86]. Therefore, each researcher is recommended to carefully select the right protocol according to their target tissue types.

**Figure 3:**
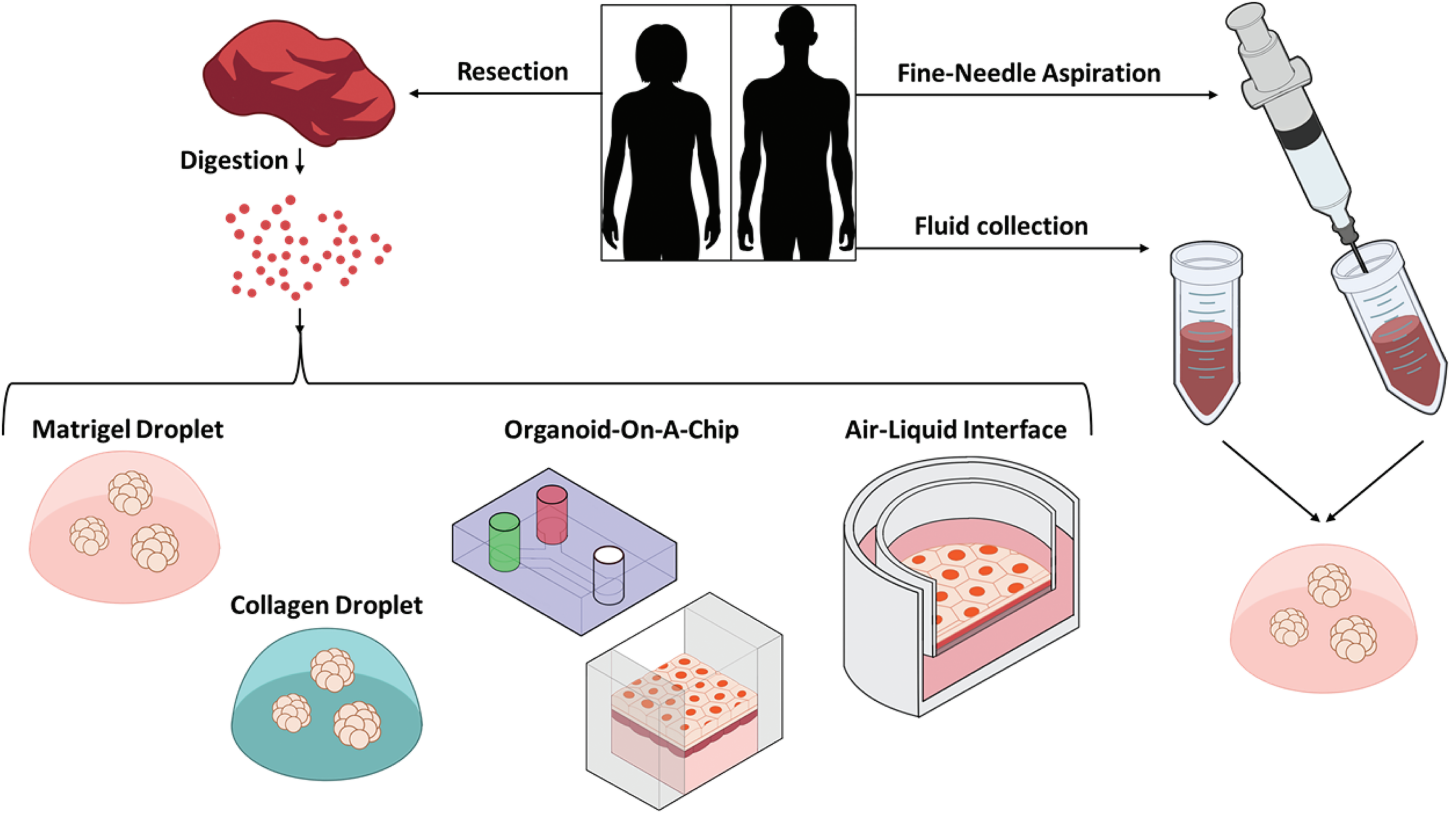
Schematic of PDO establishment methods. PDOs can be generated with or without a tissue digestion step. After tissue digestion, cells are seeded in Matrigel, Collagen, on a device, or in a chamber exposed to air. Alternatively, cells can be collected by fine-needle aspiration or from body fluid such as ascitic or pleural fluid, then seeded in Matrigel.

Here, we will summarize several methodologies for PDO generation, especially focusing on the steps following the preparation of a single-cell suspension from digested tissue. Additionally, we will mention methods that do not require tissue digestion (Fig. 3).

### Matrigel droplet protocol

Matrigel is commonly used as an ECM-mimicking scaffold. After tissue digestion, the supernatant is aspirated and the pellet is resuspended in Matrigel (mixed with a solution containing Dulbecco’s Modified Eagle Medium (DMEM)/F12, fetal bovine serum (FBS), growth factors, and antibiotics; the ratio may vary depending on protocols, but Matrigel content should be >75% in the final solution). The Matrigel mixture containing cells is then seeded as drops (~50 µL each) on the bottom of pre-heated cell culture plates (at 37°C). The plates are then transferred into a humidified incubator at 37°C for 20–30 min to let the Matrigel solidify. Some protocols suggest flipping the plate around 2–3 min after incubation starts, before complete gelation, to prevent cells from attaching to the bottom. Once the pre-warmed organoid culture medium is prepared, desired drugs may be added. Once the Matrigel drops are solidified, an organoid culture medium is added to each well. The plate is maintained in a humidified incubator at 37°C, and the medium is changed every 2&3 days by carefully aspirating and replacing it with fresh, pre-warmed medium [86,87].

### Collagen droplet protocol

An ECM-mimicking hyaluronic acid/collagen-based hydrogel is used as a scaffold. A thiolated hyaluronan/heparin component and a methacrylated collagen are mixed, and a photo-initiator is added to the solution. The resulting mixture is used to resuspend the cell pellet obtained from digested biospecimens. PTOs are formed by pipetting 5–10 μL hydrogel precursor cell suspension into plates coated with a thin layer of polydimethylsiloxane (PDMS) to help form round droplets consistently. The gels are then photo-crosslinked by ultraviolet light exposure (365 nm, 18 W cm^2^) for 1 s, initiating the crosslinking reaction through thiol-methacrylate and methacrylate–methacrylate bond formation. PTOs are subsequently maintained in cultural media. Optionally, organoids can be integrated into microfluidic devices [49,50].

### Organs-on-a-chip

Organs-on-a-chip (OOC) are microfabricated cell culture devices designed to model the functional units of human organs *in vitro* [88]. The advantages of OOC lie in their high moldability, enabling the creation of an organ-specific microenvironment *in vitro* by reproducing the basic elements or functional units essential for physiological function. For example, Huh et al. successfully reproduced the alveolar-capillary unit of the lung on an OOC by coculturing alveolar epithelial cells and lung microvascular endothelial cells. A thin and flexible PDMS microporous membrane made this co-culture possible by compartmentalizing the two different cell types. Moreover, to reproduce the deformation of the alveolar-capillary interface during breathing, the device could recreate physiological breathing movements by setting vacuum chambers on both sides of culture channels, with cyclic vacuum application inducing stretching of the cell-lined intervening membrane [89]. Mitrofanova et al. created a “gut-on-a-chip” model which enabled the formation of characteristic crypt and villus domains under dynamic systems with realistic fluid flow by combining microfabrication and hydrogel engineering [90]. While OOC models typically produced planar epithelial layers that hardly resembled the anatomical cytoarchitecture of the native gut, they overcame these limitations.

### Air-liquid interface (ALI)

The original ALI technique was established in the 1980s by Alder et al. [91] as well as Whitcutt et al. [92], and has recently been applied to organoid culture. A permeable gelatin membrane divides a culture chamber into two compartments, allowing cells to be simultaneously exposed to air and supplied with a culture medium. This setup is ideal for respiratory cells, which are supplied with medium only from the basal side while the apical surface is in contact with air, representing a more natural condition than conventional cell culture where cells are completely covered by liquid media.

Interestingly, Meindl et al. reported that differentiation of Calu-3 cells (epithelial cells from the lung) into bronchial epithelial cells with mucus production was observed only in ALI cultured cells, not in conventional liquid bathing conditions or 2D cell culture [93]. The ALI method has been applied to various cell types, such as human bronchial/tracheal and nasal epithelial cells, pancreatic cells, and intestinal cells [94–98].

Neal et al. employed the ALI method for generating PDOs using patient-derived colorectum, lung, kidney, pancreas, and thyroid cancer tumors, in combination with immunotherapy treatment [99]. The human PDO culture methodology was quite similar to previously reported methods: the cell pellet obtained from digested tumor tissue was resuspended in 1 mL of Type I collagen gel, seeded on top of pre-solidified 1 mL collagen gel within a 30, 0.4 mm inner transwell chamber, forming a double dish air-liquid culture system. The transwell containing tumor tissue and collagen was placed into an outer 60 mm cell culture dish containing 1.0 mL of culture medium. As a result, ALI PDOs typically recapitulated the histology and gene mutations of parental tumors successfully, and PDO tumor-infiltrating lymphocytes accurately preserved the original tumor T cell receptor (TCR) spectrum. Moreover, human and murine PDOs successfully modeled ICB with anti-PD-1 and/or anti-PD-L1, expanding and activating tumor antigen-specific tumor-infiltrating lymphocytes and eliciting tumor cytotoxicity. The authors speculated that organoid-based propagation will facilitate immuno-oncology investigations in the tumor microenvironment and personalized immunotherapy testing.

### No-tissue digestion protocols

Vilgelm et al. reported a unique PDO method established using melanoma, gastric cancer, colon cancer, appendiceal cancer, thyroid cancer, and renal cancer, in which enzymatic tissue digestion was not required [100]. The fine-needle biopsy technique was employed for sample collection and/or tissue disruption: using a sterile 25-gauge beveled needle attached to a sterile 10 mL syringe, the target region of the tissue was aspirated several times from different areas, helping maintain tumor heterogeneity. The collected cellular material was centrifuged, and the obtained cell pellet was reconstituted with culture solution containing DMEM/Hams F12/MCDB105, FBS, B27-supplement, and a certain percentage of Matrigel (the ratio of Matrigel may differ depending on the culture methods, between 5%–75%). As a result, the established PDO models successfully replicated the morphological features of their original tissues, such as bizarre giant cells in melanoma, signet ring cells in gastric adenocarcinoma, papillary glands in colorectal adenocarcinoma, mucin production in appendiceal cancer, microfollicular formation in thyroid cancer, and classical clear cell morphology and nested growth patterns in renal cell carcinoma.

In contrast, Choi et al. used floating cells in ascitic or pleural fluid to generate PDOs, this method also did not require tissue digestion [101]. Instead of aspirating cells from tumor tissue, 50–100 mL of ascitic or pleural fluid were collected by paracentesis or thoracentesis from patients under standard care for pancreatic, gastric, or breast cancer. The fluidic samples containing released malignant cells were centrifuged, and the obtained cell pellets were embedded into Matrigel, and supplemented with medium for cultivation. The authors successfully established PDOs from ascitic and pleural fluid originating from pancreatic, gastric, and breast cancer, demonstrating the feasibility of PDO establishment using ascites or pleural effusion.

## Patient-Derived Sarcoma Organoids (PDSOs): Applications from Carcinoma to Sarcoma Research

While most current PDOs have been optimized for carcinoma studies, some research groups have recently applied these techniques to soft tissue sarcomas, resulting in the generation of patient-derived sarcoma organoids (PDSOs). Forsythe et al. generated PDSOs from surgically resected angiosarcoma, leiomyosarcoma, gastrointestinal stromal tumor, liposarcoma, myxofibrosarcoma, dermatofibrosarcoma protuberans, and pleomorphic abdominal sarcoma using a hyaluronic acid and collagen-based ECM hydrogel [50]. In contrast, Wakamatsu et al. employed the ALI technique to generate epithelioid sarcoma PDSOs [102].

Sarcomas are a class of tumors with heterogeneous complexity, encompassing more than 60 different subtypes [103] with extremely low incidence. This makes it difficult to advance research and facilitate clinical trials, with limited success in translating preclinical discoveries into therapeutic advances. Considering these limitations, Sanchez-Fdez et al. advocated that PDSOs would be a powerful system to study patient-specific malignancies as they recapitulate an individual’s unique tumor biology [104]. Góss dos Santos claimed that his team is currently utilizing soft tissue sarcoma patient-derived models for exploring new drug applications, along with xenograft models [105].

PDSOs also have some concerns, such as the difficulty of obtaining enough biospecimens from patients who do not go for surgical resection, which could hinder the development of a broad drug array for tailor-made therapies. However, we believe that PDSOs can lead to precision cancer care in the sarcoma field. Moreover, the preclinical use of PDSOs as drug response predictors is underway, holding the hope of paving the way to personalized therapy.

## 3D Technology Applications to Liposarcoma: Up to Date

In this section, we summarize recent studies on liposarcoma using 3D techniques. We cultured five different liposarcoma cell lines using four distinct 3D cell culture methods. When scaffold-based methods were employed, Lipo141, Lipo224, Lipo815, and Lipo863 cell lines formed spheroids in Matrigel but not in Collagen. Conversely, Lipo246 did not form spheroids in either scaffold. However, this difference was not observed with scaffold-free methods, as all cell lines formed spheroids. Additionally, when treated with an MDM2 inhibitor, the 3D models showed more resistance to the drug compared to the 2D models. In conclusion, different 3D cell culture methods can significantly affect cell morphology and may enhance resistance to treatment [25,106]. We believe that understanding these methods is crucial for designing optimal 3D cell cultures for further investigations.

Liverani et al. employed the PDO technique on liposarcoma using collagen as a scaffold and injected patient-derived cells, resuspended in Matrigel, into zebrafish [107]. The 3D models exhibited higher MDM2 amplification and increased mRNA expression of β-catenin, MMP2, MMP9, and Slug, which are positively correlated with liposarcoma aggressiveness, compared to the 2D models. Moreover, patient-derived cells were successfully engrafted into zebrafish, predominantly in the body of the embryos rather than the head and tail. Forsythe et al. and Escudero et al. generated PDOs using surgically resected sarcoma tissues, including liposarcoma [50,108]. They treated PDOs with clinically used drugs to assess their efficacy for corresponding patients, highlighting the potential application of these models for personalized therapy.

## Discussion

Despite advancements in novel imaging and genomic analysis techniques, the overall survival rate of sarcoma patients has not improved in the last 30 years [50]. This stagnation is largely due to the rarity of sarcoma and a lack of understanding of the biological consequences of its tumorigenesis. Given the rarity of LPS, we believe that 3D cell culture techniques hold great promise for elucidating sarcoma features that have not yet been fully addressed.

Based on our previous results, we hypothesize that the current definition of dedifferentiated liposarcoma, characterized by the presence of excess MDM2/CDK4 through amplification or overexpression, encompasses a broad spectrum that may result in varied patient prognoses, therapeutic responses, and chemo-resistance. To better address and understand liposarcoma, we strongly advocate for a more detailed elucidation of the heterogeneity of DDLPS. Molecular characteristics revealed by multi-omics analyses are one such example. Furthermore, integrating multi-omics analyses with PDO techniques will provide new insights for preclinical discoveries and hopefully enable more accurate therapy prediction. This approach may lead to personalized therapeutic strategies, ultimately improve patient prognosis and advancing medical development.

Culturing several LPS cell lines using different 3D cell culture methods has shown that variations in 3D cell culture techniques or scaffold types can significantly affect the results and lead to different outcomes [25]. This underscores the importance of thoroughly understanding each method when planning 3D cell culture experiments. In this review, we summarized the general concepts of 3D cell culture and introduced various methods for establishing PDOs. We also discussed the application of 3D cell culture to sarcoma research, with a particular focus on liposarcoma. However, it remains difficult to select the best method depending on the specific research goal due to a lack of evidence. Currently, there are still relatively few studies focusing on DDLPS; only three papers have specifically addressed liposarcoma, with an additional two papers including liposarcoma within broader sarcoma research. This highlights the need for more focused research to elucidate the mechanisms and characteristics of DDLPS. Fortunately, the establishment and application of advanced PDO models using sarcoma tumors are already underway, as described above. We anticipate an increase in publications on this topic in the near future.

## Conclusion

To better address and understand liposarcoma, 3D cell culture represents a novel technique that we strongly advocate for in this field. PDO techniques will provide new insights for preclinical discoveries and the development of effective therapies. We have high hopes that this approach may lead to personalized therapeutic strategies, ultimately improving patient prognosis and advancing medical research.

## Data Availability

The datasets generated during and/or analyzed during the current study are available from the corresponding author on reasonable request.
